# Early Efficacy of In Situ Fenestration with a Triple Chimney Technique for High-Risk Stanford Type A Aortic Dissection: A Single-Center Retrospective Study

**DOI:** 10.1155/2021/5662697

**Published:** 2021-08-13

**Authors:** Qingsong Wu, Heng Lu, Debin Jiang, Zhihuang Qiu, Javed Rashid, Linfeng Xie, Yue Shen, Liangwan Chen

**Affiliations:** ^1^Department of Cardiac Surgery, Union Hospital, Fujian Medical University, Fuzhou, Fujian, China; ^2^Fujian Key Laboratory of Cardio-Thoracic Surgery (Fujian Medical University), Fuzhou, Fujian 350001, China; ^3^Fujian Provincial Special Reserve Talents Laboratory, Fuzhou, Fujian, China; ^4^Engineering Research Center of Tissue and Organ Regeneration, Fujian Province University, Fuzhou, Fujian, China; ^5^Fujian Medical University, Fuzhou, Fujian, China

## Abstract

**Purpose:**

The objective of this investigation was to study the early efficacy of in situ fenestration with triple chimney technique for high-risk type A aortic dissection patients.

**Methods:**

This study included 24 patients who were treated by in situ fenestration with TCT for high-risk TAAD between January 2018 and December 2019. Multiple comorbidities or preoperative critical conditions rendered patients ineligible for open surgery, but all patients that were evaluated and considered had to undergo operation. By analyzing the regular follow-up data, the early postoperative efficacy of the patients was evaluated.

**Results:**

The average age of the 24 patients was 65.4 ± 9.3 years. The success rate of the operation was 100%, as all the patients were discharged successfully. There were no serious neurological complications or persistent endoleakage. The mean follow-up time was 21.4 ± 6.9 months. The patency rate of all branching stents was 100%, with no stent displacement, stenosis, or blockage observed. While none presented with type I endoleakage, one patient (4.2%) presented asymptomatic type II endoleakage around the left subclavian artery stent. Currently, 23 of the 24 patients remain alive.

**Conclusion:**

Initial results are encouraging with TCT for high-risk TAAD. However, due to its high selectivity and potential complexity related to surgical risks, the mid- and long-term efficacy of this technique remains unknown. For patients who are eligible for open heart surgery, we still recommend it be performed.

## 1. Introduction

The development of minimally invasive endovascular aortic repair (EVAR) has been characterized through the improvement and refinement of complex surgical approaches, especially for the thoracic aorta and its branches demonstrating vascular-type pathology [1–6]. The 2014 European Society for Cardiology (ESC) Guidelines, category I, recommends type A aortic dissection (TAAD) for surgical treatment [7]. Further, a 2018, expert consensus document of the European Association for Cardio-Thoracic Surgery and the European Society for Vascular Surgery recommended surgical treatment of aortic disease involving the aortic arch [8]. However, the development of intravascular technology and thoracic endovascular aortic repair (TEVAR) with the introduction of chimney technology is changing the treatment of aortic dissection. Chimney technology involves deployment of stents/stent grafts into the supra-aortic branches, with the proximal parts connected to the main thoracic aortic endoprosthesis (between the aortic stent and the aortic wall) and extended above it to ensure perfusion. Traditional open surgery or hybrid surgery is not only traumatic and needed to restore the blood supply to the brain but also has many complications including high mortality and slow recovery after operation. It has been reported in the literature that the chimney technique was first applied to the aortic arch to preserve branching blood flow or to save the left subclavian artery from occlusion [9, 10]. At present, the use of chimney technique in preoperative planning is gradually replacing the hybrid technique in the treatment of aortic arch disease, and it has also improved in efficacy [11–14]. Treatment of a range of lesions involving the aortic arch or ascending aorta often required more proximal stent-graft placement, which covered the left common carotid, innominate arteries, or the left subclavian artery, and necessitated additional maneuvers to maintain blood flow. Alternatively, the present research examines in situ fenestration with triple chimney technique (TCT) for repair of type A aortic dissection (TAAD) in patients who were suitable but unwilling to undergo open surgery, or who were at high risk and therefore disqualified from open surgery. The aim of this single-center retrospective study was to report the results of in situ fenestration with TCT in the treatment of high-risk TAAD with early follow-up.

## 2. Methods

### 2.1. Approval

This study was approved by the institutional review board and the Department of Cardiac Surgery at Union Hospital, Fujian Medical University, Fuzhou, Fujian, P. R. China. All aspects of this study conformed to the Declaration of Helsinki.

### 2.2. Subject Cohort

**Figure 1:**
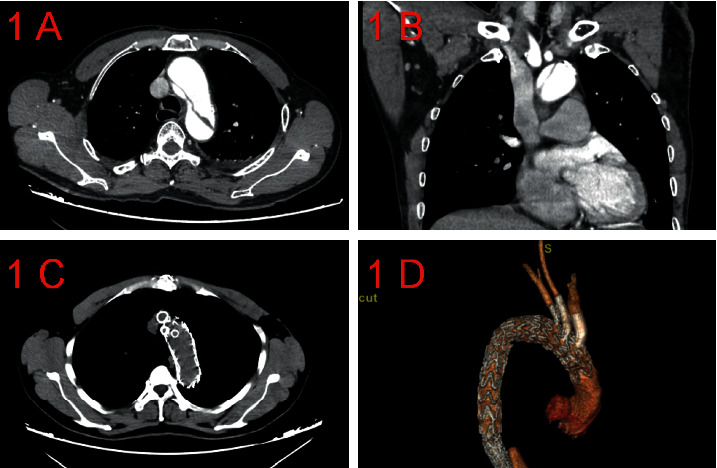
Preoperative and postoperative imaging data of acute type A aortic dissection.

**Figure 2:**
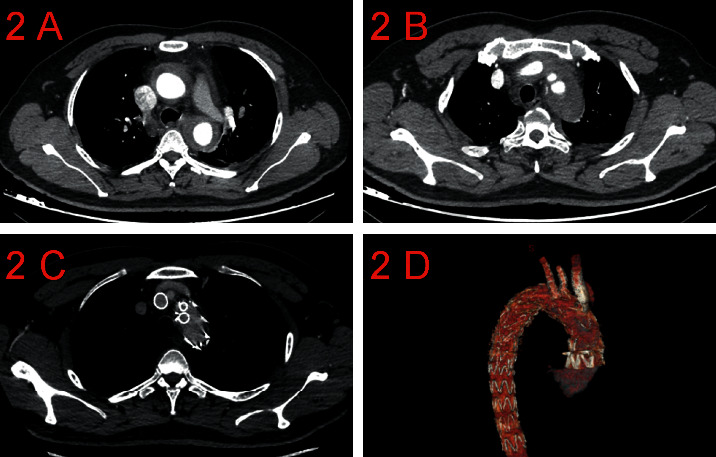
Preoperative and postoperative imaging data of chronic type A aortic dissection.

From January 2018 to December 2019, a total of 24 patients presented with TAAD, with an average age of 65.4 ± 9.3 years (range of 34–81 years), including 22 men and 22 women. There were 15 patients with acute aortic dissection (Figure 1) and nine patients with chronic aortic dissection (Figure 2). The baseline characteristics and accompanying disease of the patients are listed in Table 1. Subjects were enrolled having (1) a diagnosis of TAAD and ascending aortic size (≤40 mm) as confirmed by coronary computed tomography angiogram (CTA) and three-dimensional reconstruction and (2) if determined ineligible for conventional open surgery or hybrid surgery, following evaluation by the interdisciplinary healthcare team. After comprehensive evaluation of each subject, it was determined that in situ fenestration with TCT was appropriate treatment.

Subjects were excluded from this study if they presented with an ascending aortic size (>40 mm) and diameter of the superior branch of the aortic arch (>13 mm) that did not match the available aortic stent-graft sizes. Subjects were also excluded if indicated for other arch variants (i.e., bovine arch, etc.), or if diagnosed with the presence of aortic dissection involving the sinus of Valsalva or beyond the sinus junction with aortic valve insufficiency.

### 2.3. In Situ Fenestration with TCT Procedure

In situ fenestration with TCT was performed under general anesthesia. For patients with acute aortic dissection, analgesia and sedation were used. All patients underwent strict preoperative control of blood pressure and heart rate. Systolic blood pressure was controlled at about 90–100 mm Hg, and heart rate was 60–70 beats/min. During the operation, a 4 cm incision was made in the inguinal area to expose the femoral artery and a 5 cm incision was made in the neck along the sternocleidomastoid muscle area to expose the left common carotid artery and innominate artery. The area of the left brachial artery was disinfected, and heparin (0.5 mg/kg) was used for intravenous injection of systemic heparinization. A size 18 perfusion tube was inserted into the left femoral artery, and then size 12 perfusion tubes were inserted into the innominate and the left common carotid arteries. The connected perfusion catheter established the left femoral-innominate bypass and the left femoral-left common carotid bypass, and a piezometric catheter was connected for monitoring of blood pressure.

A pigtail catheter was inserted into the ascending aorta for angiography and to clear the location of a tear in the intimal layer of aorta. This facilitated a continuous pathway for the scope through the aortic arch and ensured a clear circulation pathway for the vertebral arteries and uninterrupted blood flow to the circle of Willis.

A large vessel-covered stent (Ankura, Lifetech Scientific Company, Ltd., Shenzhen, China) was guided through the descending aorta to the distal end of the left subclavian artery for release. A second large vessel-covered stent (Hercules, Gore & Associates, Inc., Arizona, USA) was guided to the top of the junction of the sinus of the ascending aorta and was released after accurate positioning. The distal end overlapped fully with the first large vessel-covered stent; thus, it completely covered the openings of the three branches of the aortic arch. Next, left common femoral-innominate bypass and the left common femoral-left common carotid bypass were opened. Since the left common carotid artery contained an implanted vascular sheath, a needle passing through its lumen reaches the surface of the large vessel of the covered stent at the root of the left common carotid artery. Therefore, the vascular sheath was advanced after successful puncture at the site. According to the size of each patient's blood vessels, different-sized balloon catheters were used to expand the puncture points from small to large. Puncture points were expanded until a suitable size of Viabahn stents matched the vessel (W. L. Gore & Associates, Flagstaff, AZ, USA) (size selection is bigger than original vessels; for example, 10–20% larger than the diameter of the lumen of the left common carotid artery). The end of the head was inserted into the trunk-covered stent by approximately 5–8 mm and released under fluoroscopy. The same method was used for the innominate artery covered stent insertion, thereafter inhibiting the left common femoral-innominate artery bypass and the left common femoral-left common carotid artery bypass, respectively. The left brachial artery puncture position vascular sheath was used to expand the puncture point by the third Viabahn stents in the left subclavian artery opening (head end into the trunk stent about 5–8 mm) from release under perspective, using the same aforementioned method of puncturing according to vessel size. Together, these protocols rendered a successful EVAR combined with TCT procedure.

In all patients, intraoperative angiography was used to document unobstructed blood flow, appropriate stent position, and absence of internal leakage around the stents.

After the surgical procedure, the catheter guide wire and vascular sheath were removed and hemostasis was achieved. The bilateral femoral and bilateral carotid arteries were sutured, followed by compression and binding of the brachial artery. Absence of oozing blood was verified, and skin and fascia were sutured in layers using standard technique, thus completing the operation.

### 2.4. Follow-Up

All patients had a CTA scan before discharge to evaluate the coverage of aortic lesions and the supra-aortic branch patency (Figures 1(c) and 1(d); Figures 2(c) and 2(d)). Subsequent follow-up with physical examination and CTA were scheduled at 3 months and 6 months after operation and then every 6 months, thereafter. Telephone follow-up was performed semiannually.

### 2.5. Statistical Analysis

Data are presented as mean ± standard deviation (SD); categorical data are given as the counts (percentage). Analyses were conducted using SPSS^®^ software (IBM^®^ Corporation, Somers, NY, USA). A minimum *p* value of 0.05 was considered to be statistically significant.

## 3. Results

### 3.1. Clinical Presentations

The study population included 22 men and two women, with an average age of 65.4 ± 9.3 years. All patients had TAAD, of which 11 had a history of cardiac surgery (2 patients underwent percutaneous transluminal coronary intervention, two underwent coronary artery bypass grafting, three underwent aortic valve replacement, one underwent Wheat procedure, two underwent Bentall procedure, and one underwent radiofrequency ablation). Chronic renal insufficiency with preoperative complications was presented in two cases, including one case of regular hemodialysis (3 times a week). One patient with atrial fibrillation developed hemiplegia after previous cerebral infarction, and three patients with acute aortic dissection developed shock, where one patient went into coma before surgery. Therefore, all patients underwent EVAR combined with TCT procedure, and there was no death from rupture of aortic dissection during the operation (Table 1).

### 3.2. Procedures

There were 15 cases of emergency operation, 9 cases of nonemergency operation, 3 cases of preoperative shock, and one case of preoperative coma for emergency operation. All operations were successfully completed, as there was no case of death during intraoperative aortic rupture massive hemorrhage, except in the case of one patient with preoperative chronic renal failure, who underwent postoperative hemodialysis. Nonetheless, postoperative angiography showed that all stents were unobstructed without internal leakage. In all subjects, the circle of Willis was found to be fulfilled during the operation. The dominant type of left vertebral artery was confirmed by angiography in four cases, but there was no presentation of left subclavian artery steal syndrome in any study subject. The total time for the operative procedure time was 237.4 ± 40.6 minutes in all patients, and fluoroscopy time and volume of contrast media (iohexol injection) (GE Healthcare Ireland) were 68.8 ± 10.4 minutes and 171.3 ± 23.2 ml, respectively.

### 3.3. Morbidity and Mortality

Average postoperative ICU stay was 1.8 days (1.8 ± 1.1 days) and the average length of stay was 15.5 days (15.5 ± 7.1 days). Following surgery, 5 patients had transient fever with the highest temperature of 38.6°C and the longest duration of 40 hours. No internal leakage was found after the routine thoracic aorta CTA reexamination before discharge, and no sequelae or death occurred in all patients (Table 2).

### 3.4. Follow-Up

During the follow-up, for an average of 21.4 ± 6.9 months, subjects were prescribed regular oral aspirin (100 mg/d). No severe neurological complications such as paraplegia or stroke were observed during the follow-up, and no left subclavian artery steal blood syndrome, left upper limb ischemia, leakage around the stent, stent displacement or discontinuity, stent stenosis, nor blockage were found. One patient with Marfan syndrome underwent abdominal aortic stent implantation due to residual aortic dissection and small true lumen 3 months after the operation, but unfortunately, the operation failed and this patient died. During the entire follow-up period, the chimney stent remained unobligated in all cases, and all patients survived, except for the aforementioned patient with Marfan syndrome (Table 3).

## 4. Discussion

Conventional treatment of TAAD requires open surgery with extracorporeal circulation at low temperature. However, this treatment is associated with a high mortality rate and many complications, which remain high despite improvements in methods of open surgical treatment of aortic dissection [15, 16]. Currently, TEVAR is considered to be a less invasive and more effective method for aortic lesions involving the aortic arch for high-risk patients. Chimney technology, including large vessel stents implanted into a small covered stent, has been widely used in aortic dissection involving the left subclavian artery; otherwise, the aortic arch rivet distance is insufficient. This not only preserved the blood supply to the important branches covered by the aortic stent but also provided more landing sites for the main arterial stent. This technique reduces the risk of cerebral ischemia and has been shown to have good midterm efficacy [17, 18]. Compared with traditional surgery, endovascular therapy is a novel intervention that is minimally invasive and safer for the treatment of aortic lesions [19, 20].

All patients with aortic dissection are suitable for open surgery, mainly because of poor preoperative health. Therefore, the present study used multibranch stent implantation to accommodate these patients [21]. However, due to the limitations of operating difficulty, the number of study subjects is small in the research herein. Further, the implantation of stents on the aortic arch would have increased the complexity of the operation, and the instability of multiple grafts would have prolonged operation time, thus increasing the risk during the operation. In addition, radiation time for subjects and the volume of contrast agent used would have increased, as well.

In this study, all subjects had covered stents for their supra-aortic branches. The advantage of covered stents is the capacity to reduce the occurrence of internal leakage; however, they increased the difficulty of operation. Because of this, it was required to open the trunk-covered stent at the root of branch vessels by the needle puncture and then expand the puncture point of the trunk-covered stent with a balloon. Once punctured, we needed to accurately locate the center of the trunk-covered stent between the two metal areas so that the Viabahn-covered stents would be better matched for the expanded hole of trunk-covered stent. This method can reduce leakage around the covered stent, and the trunk stents can be compatible with their own blood vessels, so as to avoid their rupture in the residual dissection, avoiding thrombosis or the occurrence of plaque detachment, and subsequent cerebral infarction.

It has been reported in the article that the chimney stent technology used bare stents, which could avoid affecting the blood flow of the trunk stent, but there was a risk of type Ia internal leakage. In 2006, Hiramoto et al. first reported the use of covered stents in the chimney technology to reduce the incidence of internal leakage [22], like in the present study. The technique used here extended the Viabahn stent 5–8 mm into the trunk-covered stent to form a chimney shape. This helped match the original branch vessels with the stent vessels and avoid internal leakage due to stent disassembly or uneven force loading. However, if the vessel of the branching stent extends into the trunk stent deep in length, then aortic blood flow may impact the stent to form shear force, resulting in branch stent displacement and internal leakage. If the insertion distance is insufficient, it might lead to the shifting and deformation of the branch stent due to the insufficient rivet distance, causing internal leakage, narrowing, or breakage of the stent. At the same time, the branching covered stent can enter the lumen of the trunk stent and form an angle with the direction of blood flow, which may lead to insufficient blood pressure in the stent and related complications due to poor cerebral perfusion. It may also lead to slower blood flow in the stent, causing platelet aggregation and leading to stenosis and blockage of the branching covered stents. During subject follow-up in the present study, there were no cases of stent displacement, deformation, stenosis, or leakage around the stents. This is due to the suitable placement of the branch-covered stents, as they were in the same direction as the original aortic arch vessels. Decreased perfusion was not documented likely secondary to the three branch-covered stents that opened cylindrically, resulting in the lowest shear force in blood flow. By stretching out the length of the trunk, stenting blood vessels was appropriate and the three branch-covered stents supported joint stress, allowing the blood flow sheared force uniform distribution (i.e., vessel deformation from strain) and enabling stabilization of the branch-covered stents.

During follow-up, asymptomatic type II internal leakage around the left subclavian artery stent was found in one case, which was likely caused by the formation angle between the left subclavian artery stent and the main stent. The blood pressure was very high in the aorta, and when the blood flow passed through the left subclavian artery, the angle between the left subclavian artery and the aortic-covered stent was too small, resulting in excessive pressure in the root of the left subclavian artery and blood passing through the main covered stent to form a type II internal leakage. The solution here involved employing stents that were 10–20% greater than the original vessels, such that branch-covered stents may fit the original artery blood wall well. Thus, when the left subclavian artery and aorta angle was too small, slightly larger branch-covered stents and smaller balloons were used in subjects. Balloon expansion was allowed in the left subclavian artery blood flow on the basis of guarantee that maximum expansion kept the trunk's blood support intact in the left subclavian artery stent, maintaining impermeability and stability of the roots.

While TCT for surgeons demands a high level of technology, due to considerable technical difficulty and with some potential risks, the long-term effect remains unknown. Although further follow-up observations are required, this method can still be used to save those who do not have many surgical options such as those who are critically ill or patients at high risk of surgical complications. For some patients with dominant right vertebral artery and a complete patent circle of Willis, the double chimney technique of innominate artery and left common carotid artery stent implantation can be performed. Left subclavian artery stent implantation was abandoned by the present research team to reduce the time and difficulty of surgery, hence also reducing the occurrence of potential left subclavian artery steal syndrome [4, 23]. However, this may increase the occurrence of type II leakage in the root of the left subclavian artery [24]. The solution to this problem has been reported, where the left subclavian artery orifice can be blocked with an arterial catheter occluding device [23], but this was unnecessary in the present work because type II leakage was not observed in subjects during procedures. Simultaneously, it may reduce the risk of paraplegia and other unknown risks in TEVAR. While the long-term efficacy of double chimney still remains uncertain, the results of this study suggest a preference for TCT as far as the patient's condition allows.

### 4.1. Limitations

This study is a single-center retrospective study with a small sample size. The follow-up time is short, as the mid- and long-term results are still underway. All the included cases have high selectivity, which is not applicable to all the patients. Moreover, the surgical operation requires an elite skillset by the surgeon, making the popularization of this surgical technique a challenge. The technique also has the potential to reverse the dissection of the ascending aorta during surgery and even lead to rupture and death.

## 5. Conclusion

Initial results are encouraging for EVAR combined with TCT for high-risk TAAD. However, due to its high selectivity to patients, potential complexity, and surgical risks, the medium- and long-term efficacy of this technique is still unknown, and it should be used with caution.

## Figures and Tables

**Table 1:** Demographic characteristics of the subject cohort.

Projects	Results
Age (years)	65.4 ± 9.3
Male gender, *n* (%)	22 (91.7)
BMI (kg/m^2^)	25.7 ± 3.6
Acute aortic dissection, *n* (%)	15 (62.5)
Hypertension, *n* (%)	23 (95.8)
Marfan syndrome, *n* (%)	1 (4.2)
Coronary heart disease, *n* (%)	5 (20.8)
Diabetes mellitus, *n* (%)	3 (12.5)
Chronic renal failure, *n* (%)	2 (8.3)
Chronic obstructive pulmonary disease, *n* (%)	2 (8.3)
Cardiac operation history, *n* (%)	11 (45.8)
Atrial fibrillation, *n* (%)	4 (16.7)
Neurological sequelae, *n* (%)	3 (12.5)
Shock, *n* (%)	3 (12.5)
Coma, *n* (%)	1 (4.2)
Heart function classification (NYHA)	
II	4 (16.7)
III	16 (66.7)
IV	4 (16.7)
Ejection fraction (%)	58.5 ± 9.6
Emergency operation, *n* (%)	15 (62.5)

Continuous data are presented as the means ± standard deviation; categorical data are given as the counts (percentage).

**Table 2:** Operative and postoperative data of the study subjects.

Projects	Results
Volume of contrast media (ml)	171.3 ± 23.2
Fluoroscopy time (mins)	68.8 ± 10.4
Procedure time (mins)	237.4 ± 40.6
ICU stay time (days)	1.8 ± 1.1
Length of stay (days)	15.5 ± 7.1
Fever, *n* (%)	5 (20.8)
Left subclavian artery steal syndrome, *n* (%)	0 (0.0)
Left upper limb ischemia, *n* (%)	0 (0.0)
Early leakage, *n* (%)	0 (0.0)
Early death, *n* (%)	0 (0.0)
Early spinal cord injury, *n* (%)	0 (0.0)
Permanent nervous system injury, *n* (%)	0 (0.0)
Transient nervous system symptom, *n* (%)	0 (0.0)
Acute kidney injury, *n* (%)	0 (0.0)

Continuous data are presented as the means ± standard deviation; categorical data are given as the counts (percentage).

**Table 3:** Follow-up data on study subjects.

Projects	Results
Follow-up time (months)	21.4 ± 6.9
Follow-up death, *n* (%)	1 (4.2)
Reinterventions, *n* (%)	0 (0.0)
Late endoleakage, *n* (%)	1 (4.2)
Stent displacement, *n* (%)	0 (0.0)
Stent stenosis, *n* (%)	0 (0.0)
Stent blockage, *n* (%)	0 (0.0)

Continuous data are presented as the means ± standard deviation; categorical data are given as the counts (percentage).

## Data Availability

All data generated or used during the study appear in the submitted article.

## Abbreviations

CTA: Coronary computed tomography angiogram
EVAR: Endovascular aortic repair
ESC: European Society for Cardiology
SD: Standard deviation
TEVAR: Thoracic endovascular aortic repair
TCT: Triple chimney technique
TAAD: Type A aortic dissection.

## References

[1] Nienaber C. A., Kische S., Rousseau H. (2013). Endovascular repair of type B aortic dissection. *Circulation: Cardiovascular Interventions*.

[2] Yang J., Xiong J., Liu X., Jia X., Zhu Y., Guo W. (2012). Endovascular chimney technique of aortic arch pathologies: a systematic review. *Annals of Vascular Surgery*.

[3] Sugiura K., Sonesson B., Akesson M., Björses K., Holst J., Malina M. (2009). The applicability of chimney grafts in the aortic arch. *The Journal of Cardiovascular Surgery*.

[4] Chang S., Luo M._Y., Li Q._M. (2011). Early result of left carotid chimney technique in endovascular repair of acute non-a-non-b aortic dissections. *Journal of Endovascular Therapy*.

[5] Zimpfer D., Czerny M., Kettenbach J. (2006). Treatment of acute type a dissection by percutaneous endovascular stent-graft placement. *The Annals of Thoracic Surgery*.

[6] Metcalfe M. J., Holt P. J., Hinchliffe R. J., Morgan R., Loftus I. M., Thompson M. M. (2012). Fenestrated endovascular aneurysm repair: graft complexity does not predict outcome. *Journal of Endovascular Therapy*.

[7] Erbel R., Aboyans V. (2014). 2014 ESC Guidelines on the diagnosis and treatment of aortic diseases. *European Heart Journal*.

[8] Czerny M., Schmidli J., Adler S. (2019). Editor’s choice - current options and recommendations for the treatment of thoracic aortic pathologies involving the aortic arch: an expert consensus document of the European association for cardio-thoracic surgery (EACTS) & the European society for vascular surgery (ESVS). *European Journal of Vascular and Endovascular Surgery*.

[9] Ohrlander T., Sonesson B., Ivancev K., Resch T., Dias N., Malina M. (2008). The chimney graft: a technique for preserving or rescuing aortic branch vessels in stent-graft sealing zones. *Journal of Endovascular Therapy: An Official Journal of the International Society of Endovascular Specialists*.

[10] Criado F. J., Barnatan M. F., Rizk Y. (2002). Technical strategies to expand stent-graft applicability in the aortic arch and proximal descending thoracic aorta. *Journal of Endovascular Therapy*.

[11] Hogendoorn W., Schlösser F. J. V., Moll F. L., Sumpio B. E., Muhs B. E. (2013). Thoracic endovascular aortic repair with the chimney graft technique. *Journal of Vascular Surgery*.

[12] Zhu Y., Guo W., Liu X., Jia X., Xiong J., Wang L. (2013). The single-centre experience of the supra-arch chimney technique in endovascular repair of type B aortic dissections. *European Journal of Vascular and Endovascular Surgery*.

[13] Baldwin Z. K., Chuter T. A. M., Hiramoto J. S., Reilly L. M., Schneider D. B. (2008). Double-barrel technique for endovascular exclusion of an aortic arch aneurysm without sternotomy. *Journal of Endovascular Therapy*.

[14] Wang T., Shu C., Li M. (2017). Thoracic endovascular aortic repair with single/double chimney technique for aortic arch pathologies. *Journal of Endovascular Therapy*.

[15] Kurazumi H., Mikamo A., Kudo T. (2014). Aortic arch surgery in octogenarians: is it justified?. *European Journal of Cardio-Thoracic Surgery*.

[16] Minakawa M., Fukuda I., Yamauchi S. (2010). Early and long-term outcome of total arch replacement using selective cerebral perfusion. *The Annals of Thoracic Surgery*.

[17] Tsilimparis N., Debus E. S., von Kodolitsch Y. (2016). Branched versus fenestrated endografts for endovascular repair of aortic arch lesions. *Journal of Vascular Surgery*.

[18] Spear R., Haulon S., Ohki T. (2016). Editor’s choice-subsequent results for arch aneurysm repair with inner branched endografts,. *European Journal of Vascular and Endovascular Surgery*.

[19] Fattori R., Tsai T. T., Myrmel T. (2008). Complicated acute type B dissection: is surgery still the best option?. *JACC: Cardiovascular Interventions*.

[20] Beropoulis E., Fazzini S., Austermann M., Torsello G. B., Damerau S., Torsello G. F. (2020). Long-term results of thoracic endovascular aortic repair using a low-profile stent-graft. *Journal of Endovascular Therapy*.

[21] Schumacher H., Böckler D., Bardenheuer H., Hansmann J., Allenberg J.-R. (2003). Endovascular aortic arch reconstruction with supra-aortic transposition for symptomatic contained rupture and dissection: early experience in 8 high-risk patients. *Journal of Endovascular Therapy*.

[22] Hiramoto J. S., Schneider D. B., Reilly L. M., Chuter T. A. M. (2006). A double-barrel stent-graft for endovascular repair of the aortic arch. *Journal of Endovascular Therapy*.

[23] Liu H., Shu C., Li X. (2015). Endovascular aortic repair combined with chimney technique in the treatment of Stanford type B aortic dissection involving aortic arch. *Annals of Vascular Surgery*.

[24] Criado F. J., Barnatan M. F., Rizk Y., Clark N. S., Wang C. F. (2002). Technical strategies to expand stent-graft applicability in the aortic arch and proximal descending thoracic aorta. *Journal of Endovascular Therapy*.

